# Mining Relational Paths in Integrated Biomedical Data

**DOI:** 10.1371/journal.pone.0027506

**Published:** 2011-12-06

**Authors:** Bing He, Jie Tang, Ying Ding, Huijun Wang, Yuyin Sun, Jae Hong Shin, Bin Chen, Ganesh Moorthy, Judy Qiu, Pankaj Desai, David J. Wild

**Affiliations:** 1 School of Library and Information Science, Indiana University, Bloomington, Indiana, United States of America; 2 School of Computing and Informatics, Indiana University, Bloomington, Indiana, United States of America; 3 Department of Computer Science and Technology, Tsinghua University, Beijing, China; 4 School of Pharmacy, University of Cincinnati, Cincinnati, Ohio, United States of America; Wayne State University, United States of America

## Abstract

Much life science and biology research requires an understanding of complex relationships between biological entities (genes, compounds, pathways, diseases, and so on). There is a wealth of data on such relationships in publicly available datasets and publications, but these sources are overlapped and distributed so that finding pertinent relational data is increasingly difficult. Whilst most public datasets have associated tools for searching, there is a lack of searching methods that can cross data sources and that in particular search not only based on the biological entities themselves but also on the relationships between them. In this paper, we demonstrate how graph-theoretic algorithms for mining relational paths can be used together with a previous integrative data resource we developed called Chem2Bio2RDF to extract new biological insights about the relationships between such entities. In particular, we use these methods to investigate the genetic basis of side-effects of thiazolinedione drugs, and in particular make a hypothesis for the recently discovered cardiac side-effects of Rosiglitazone (Avandia) and a prediction for Pioglitazone which is backed up by recent clinical studies.

## Introduction

The emerging fields of *chemogenomics* [1] and *systems chemical biology* [2] require examination of critical associations between individual entities (genes, compounds, etc). Identification of semantic associations can utilize many of the methods of graph theory, such as finding shortest paths between entities, and along with Semantic Web methods forms the basis of our work here. However, the complex structure of the ontologies involved, the heterogeneity of the data sources, and sheer size of some of the datasets make this a non-trivial problem: one requires a highly efficient and scalable framework to identify semantic associations in the biomedical field. Additionally, there are usually many linked paths between two instances; thus providing contextual evaluation of those different linked paths is also a critical problem.

The Semantic Web provides machine-understandable semantics for resources, establishing a common platform to integrate heterogeneous data sources, and tools for searching and data mining these sources in an integrative fashion. Semantic Web methods have been adopted in various areas of life sciences, healthcare, and drug discovery [3]–[4], through various projects including Chem2Bio2RDF (developed in our labs) [5], Bio2RDF [6], Linking Open Drug Data (LODD) project [7], and Linked Life Data, which convert data to a common syntax and specify the meaning of the data through formal, logic-based ontologies or schemas. In particular, discovering and ranking complex links and relationships between resources are critical steps toward knowledge discovery. In the biomedical domain, there is a vital need for cross-domain data mining. Recent technological and experimental advances in genomics, compound screening in particular have resulted in an explosion of public data of chemical compounds, drugs, genomes, biological molecules, and in scholarly publications that pertain to these entities. Consequently, new informatics-based integrative domains have emerged, including cheminformatics [8], chemogenomics [1] and systems chemical biology [2]. Cheminformatics pertains to the large-scale analysis of chemical structures and their relationships to biological entities; chemogenomics to the relationships between chemical compounds and genes or protein targets, and systems chemical biology to the system-wide application of these techniques (where the system is a cell or organism as a whole).

In this paper, we first describe an algorithm for tackling this: a scalable path finding algorithm that works on RDF (the basis on describing relationships in the Semantic Web) and an algorithm based on LDA [9] which we call Bio-LDA, that extracts topics from large quantities of biomedical literature and gives the probabilistic distribution of biological terms (e.g., compounds, diseases, and genes) among different topics, so as to provide contextual information for those identified semantic associations. Through the integration of the path finding algorithm and a Bio-LDA algorithm we have developed for ranking paths using literature associations [10] with our prior work to develop an integrated RDF systems chemical biology resource [5], we demonstrate how important semantic and literature-contextualized paths can be identified and evaluated. We discuss this process using two biomedical case studies.

In the context of Semantic Web as a whole, the problem of discovering and reasoning complex relationships between resources has been studied by many researchers, most of which studied a specific subset of such relationships, or relationships that bear certain properties. Anyanwu et al. [11]–[13] originally formalized an important subset of complex relationships called Semantic Associations that are mainly based on undirected or directed paths. Anyanwu et al. [11], [13] define three types of complex relationships based on Property Sequence (PS) that is a finite sequence of properties defined in RDFS: *ρ – Path* association capturing the connectivity feature between two resource; *ρ – Join* association indicating that resources *r_1_* and *r_2_* relate to the same resource; *ρ – ISO* association identifying the similarity between *r_1_* and *r_2_*. A following-up work [13] formalized the definition of semantic associations and presented outlines of two implementations of *ρ – operator*. The first approach is to build a separate *ρ – query* processing layer from a storage system. The *ρ – query* processing layer maintains an index called PathGuide that keeps the path information among classes extracted from schema. However, this is not very scalable when a large index size and number of queries for validation is needed. The second approach is to use graph algorithms on memory-resident RDF graphs. However, the RDF graphs are usually too large to fit into memory. Sheth et al. [14] combined novel academic research and commercialized semantic web technology to provide capabilities of semantic association identification. Faloutsos et al. proposed an algorithm to identify an informative subgraph between two nodes [15]. Mulla et al. proposed three heuristics to calculate weights of edges and assigned weights to edges of the RDF graph [16] and applied the algorithm proposed in [15]. Perry et al. introduced a system for computing Semantic Associations over distributed RDF data stores in a peer-to-peer setting [17]. For semantic association finding in the biomedical domain, Dong et al. described a prototype system for mining the semantic associations in ontology structure and search for instances that belong to the nodes and edges along the identified path through SPARQL [18].

Another approach to the discovery of semantic association is to use a query language that supports semantic association queries. Kochut and Janik [19] present SPARQLeR, a novel extension of the SPARQL query language which adds the support for semantic path queries. The proposed extension fits seamlessly within the overall syntax and semantics of SPARQL and allows easy and natural formulation of queries involving a wide variety of regular path patterns in RDF graphs. SPARQLeR's path patterns can capture many low-level details of the queried associations. Other similar studies include SPARQ2L, PSPARQL (path RDF query language) [20].

In the field of topic identification and text mining, since Blei et al. [9] introduced the LDA model, various extended LDA models have been used in automatic topic extraction from text corpora. LDA and its extended models have been broadly used in many areas including the biomedical domain. Zheng et al. [21] applied the classic LDA model to protein-related MEDLINE titles and abstracts and extracted 300 major topics. They further mapped those topics to Gene Ontology (GO) terms. Blei et al. [22] examined 5,225 free-text items in the Caenorhabditis Genetic Center (CGC) Bibliography using the classic LDA model. They found that the LDA model had better predictive performance than two standard models (unigram and mixture of unigrams) trained using the same data. Bundschus et al. [23] presented a Topic-Concept model, which extends the basic LDA framework to reflect the generative process of indexing a PubMed abstract with terminological concepts from an ontology.

In this paper, we propose a scalable path finding algorithm that can not only detect paths between instances belonging to different classes but also between instances belonging to the same class. In addition, we complement the algorithm with a Bio-LDA model which extracts contextual information on topics of bio-terms, which helps to evaluate and interpret the semantic associations. This paper is organized as follows: Section 2 describes the materials and methods; Section 3 presents the results, including two case studies; section 4 presents a discussion of the results.

## Materials and Methods

### 2.1 Datasets

The work reported in this paper uses the *Chem2Bio2RDF* resource [5]. Chem2Bio2RDF covers 25 biomedical datasets, grouped into 6 domains, namely chemical (PubChem Compound, ChEBI, PDB Ligand), chemogenomics (KEGG Ligand, CTD Chemical, BindingDB, MATADOR, PubChem BioAssay, QSAR, TTD, DrugBank, ChEMBL, Binding MOAD, PDSP, PharmGKB), biological (UNIPROT, HGNC, PDB, GI), systems (KEGG Pathway, Reactome, PPI, DIP), phenotype (OMIM, Diseasome, SIDER, CTD diseases) and literature (MEDLINE/PubMed). At the time of writing, the numbers of triples (i.e. relationships encoded) is about 78 million. Provenance information has been added and the data has been linked to LODD and Bio2RDF [6] using *owl:sameAs* constructs.

Additionally, biological terms that are found in these datasets (compounds, drugs, genes, diseases and side-effects; collectively we call these *BioTerms*) are identified in scholarly journal abstracts in PubMed, and these terms are used to link Publications (as identified by a PubMed ID) with entries in Chem2Bio2RDF datasets. The BioTerm PubMed-dataset relationships are converted to RDF triples and integrated with Chem2Bio2RDF. Table 1 gives some statistics on the extracted BioTerms. The data schema used in our system is designed based on the category of bio-terms (compound, drug, gene, disease, side effect, pathway) and DTD (Document Type Definition) provided by National Library of Medicine (NLM). Bio-term dictionaries are generated from the following data sources listed in Chem2Bio2RDF: the compound dictionary is generated from PubChem Synonym with the PubChem Compound identifier (CID); the drug dictionary is generated from DrugBank and used DBID as the identifier; the gene dictionary is generated from the HGNC and used UniprotID as the identifier; the disease dictionary is generated from the CTD (the comparative toxicogenomics database) and used MeshID as the identifier; the side effect dictionary is generated from the Sider and used UMLSID as the identifier; the pathway dictionary is generated from the KEGG pathway and used KeggID as the identifier. We parsed the XML file and extracted the terms based on the pre-generated dictionaries.

**Table 1:** Statistics of the bio-terms extraction.

Bio-Terms	# of unique terms	# of term-citation pairs	# of unique citations
Compound	56,383	11,775,891	5,856,084
Drug	2,820	5,624,529	3,427,067
Gene	13,022	5,252,844	3,735,517
Disease	3,848	12,612,636	7,066,084
Side Effect	1,363	10,489,676	6,310,741
Pathway	180	916,754	838,090

### 2.2 Algorithm for Pathfinding in RDF data

We have developed a scalable and efficient path finding algorithm that is designed to find all of the paths between any two entities in the RDF network. In the area of network analysis, the task of association search can be formalized as a task of path search in the graph. Algorithms for shortest path [24]–[25], efficient shortest paths in sparse networks [26], top-k shortest paths [27]–[28], and near-shortest paths [29] have been proposed. See [30]–[32] for overviews. See also [33]. The algorithms for shortest path have been applied to, for instance, find the best routines of vehicles or messages, find optimal flows in networks (treated for example in [34]) and traffic-light networks [35], and find the k most likely state sequences from the HMM graph given the observed acoustic data [36].

We are given a semantic network (e.g., Chem2Bio2RDF), which can be represented as a graph *G* = (*V*, *E*), where *v*∈*V* represents an entity in the network; *e^r^_ij_*∈*E* represents a relationship with property *r* (e.g., drug interaction) between entities *v_i_* and *v_j_*; the relationship can be directional or bi-directional; the goal of association is to find relationship sequences from *v_i_* to *v_j_*. The association here is defined as: *Given a network G* = (*V*, *E*), *the association α*(*v_i_*, *v_j_*) *is a sequence of relationships* {*e^r^_i_*_1_, *e^r^*_12_, …, *e^r^_lj_*} satisfying *e^r^_m_*_(*m*+1)_∈*E* for *m* = 1, 2, …, *l*−1, where *v_i_ and v_j_ are the source entity and the target entity, respectively.*

We assume that no entity will appear on a given association more than one time. We define the process of association search from one entity to the other as: *Given an association query* (*v_i_*, *v_j_*), *where v_i_ denotes the source entity and v_j_ denotes the target entity. Association search is to find possible associations* {*α_k_*(*v_i_*, *v_j_*)} *from v_i_ to v_j_*.

In this paper, we formalize the association search problem as that of near-shortest associations. We use a two-stage approach for finding the near-shortest associations. The input is an association query (*v_i_*, *v_j_*). The objective is to find list of associations *A*(*v_i_*, *v_j_*) = {*α_k_*(*v_i_*, *v_j_*)}.

By combining the initialization step and the output step, our approach consists of four steps:

1. Initialization. We formalize the network as a directed graph. We view each entity as a node and each relationship as an edge in the directed graph. We create an index for the directed graph and load the index into memory for the following steps.
2. Shortest association finding. It aims at finding the shortest associations from all entities *v*∈*V*\*v_j_* in the network to the target entity *v_j_* (including the shortest association from *v_i_* to *v_j_* with length *L_min_*). In a graph, the shortest path between two nodes can be found using the state-of-the-art algorithms, for example, Dijkstra algorithm. However, we are dealing with a large-scale network, where the conventional Dijkstra algorithm results in a high time complexity of *O*(*n* ^2^). We propose using a heap-based Dijkstra algorithm to quickly find the shortest associations that can achieve a complexity of *O*(*n*log*n*).
3. Near-shortest associations finding. Based on the length of shortest association *L_min_* found in Step 2 and a pre-defined parameter *β*, the algorithm requires enumeration of all associations that are less than (1+*β*)*L_min_* by a depth-first search. We constrain the length of an association to be less than a pre-defined threshold. This length restriction can reduce the computational cost.

**Figure 1:**
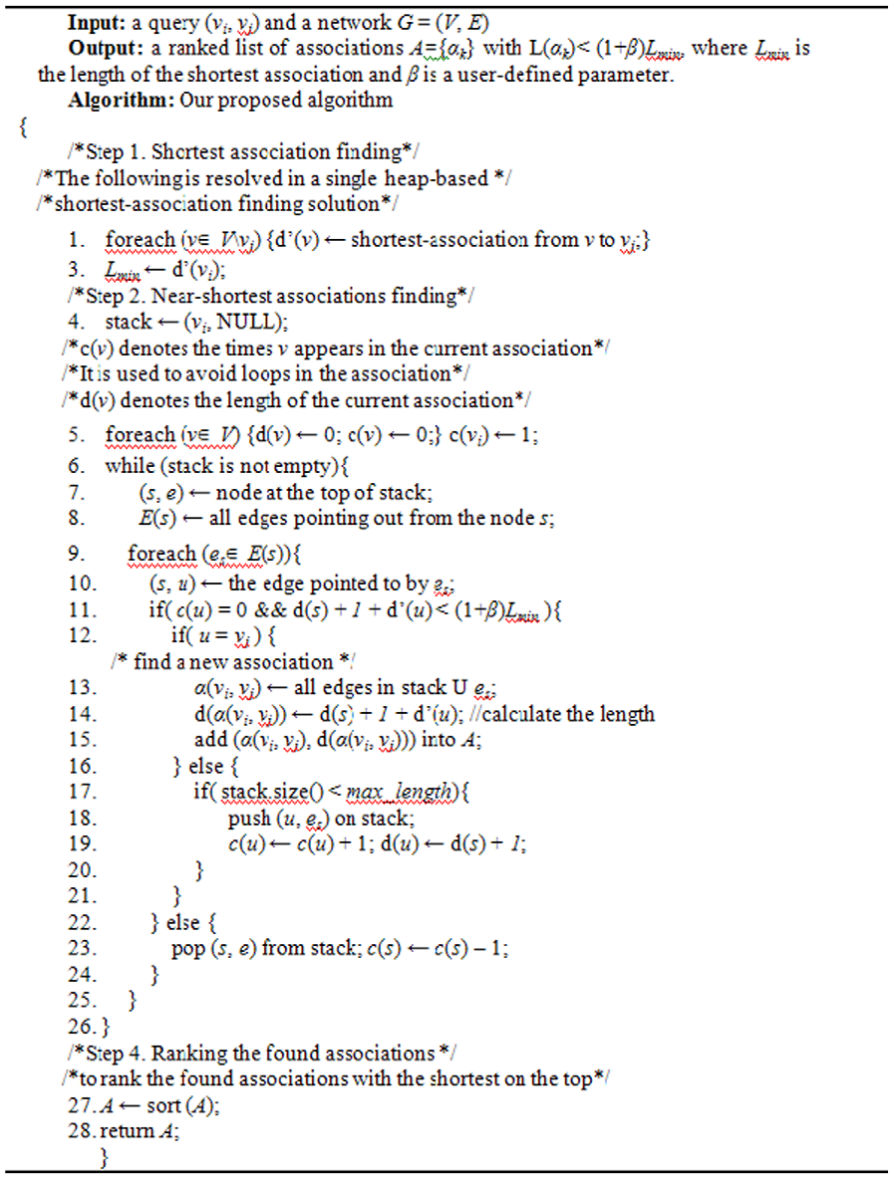
Shortest path algorithm. The pseudo code for the shortest path finding algorithm.

The correctness of the approach follows from the obvious dynamic programming interpretation of Step 2 and Step 3. Figure 1 summarizes the proposed algorithm. In the rest of the section, we will explain the two main stages (Step 2 and Step 3).

### 2.2.1 Algorithm for Shortest Association Finding

**Figure 2:**
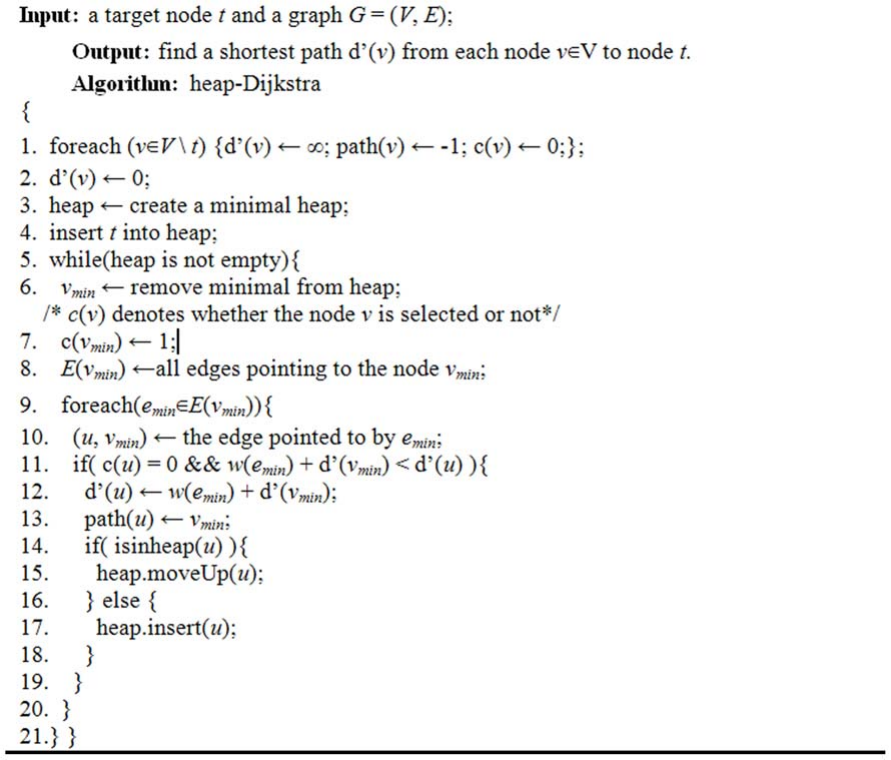
Heap-Dijkstra algorithm.

In the second step of the approach, we try to find the shortest associations from all entities (*v*∈*V*\*v_j_*) to the target entity *v_j_*. The step is necessary a_s all of the found shortest associations d′(*v_i_*) will be used to guide the search process in Step 3. Dijkstra is the traditional approach for the shortest path search in a graph; however, the conventional Dijkstra algorithm has a complexity of *O*(*n* ^2^), making it inefficient for a large graph. We use a heap-based Dijkstra algorithm (*heap-Dijkstra*) which has a complexity of *O*(*n*log(*n*)). The heap-Dijkstra is summarized in Figure 2.

In the heap-Dijkstra algorithm, we firstly create a minimal heap. Then, in each iteration of the algorithm, we use the heap to find the minimal value. The function is in heap() in line 14 is to determine whether the node *u* has been inserted into the heap or not. The operations “moveUp” and “insert” are respectively used to resort the heap and to insert a node into the heap. This focuses on finding the shortest path from each node to a specified target node. This is different from the traditional use of the Dijkstra algorithm where the objective is usually to find the shortest path from a specified source node to each of the other nodes. We conducted complexity analysis of the algorithm. As all nodes may be inserted into heap, the complexity of the loop from line 5 is *O*(*n*). In the loop, the algorithm requires enumerating all edges *E*(*v_min_*) pointing to the selected node *v_min_*. Usually, we have |*E*(*v_min_*)|≪|*V*|, where |*E*(*v_min_*)| is the number of edges pointing to the node *v_min_* and |*V*| is the number of nodes in graph *G*. In our research network, the average number of edges pointing to a node is about 5. Hence, we view the complexity of the loop in line 9 as *O*(1). The running time of the operation “moveUp” in line 15 is log(*n*), necessitating the operation “insert” in line 17. Therefore, the final complexity of the algorithm is *O*(*n*log(*n*)).

**Figure 3:**
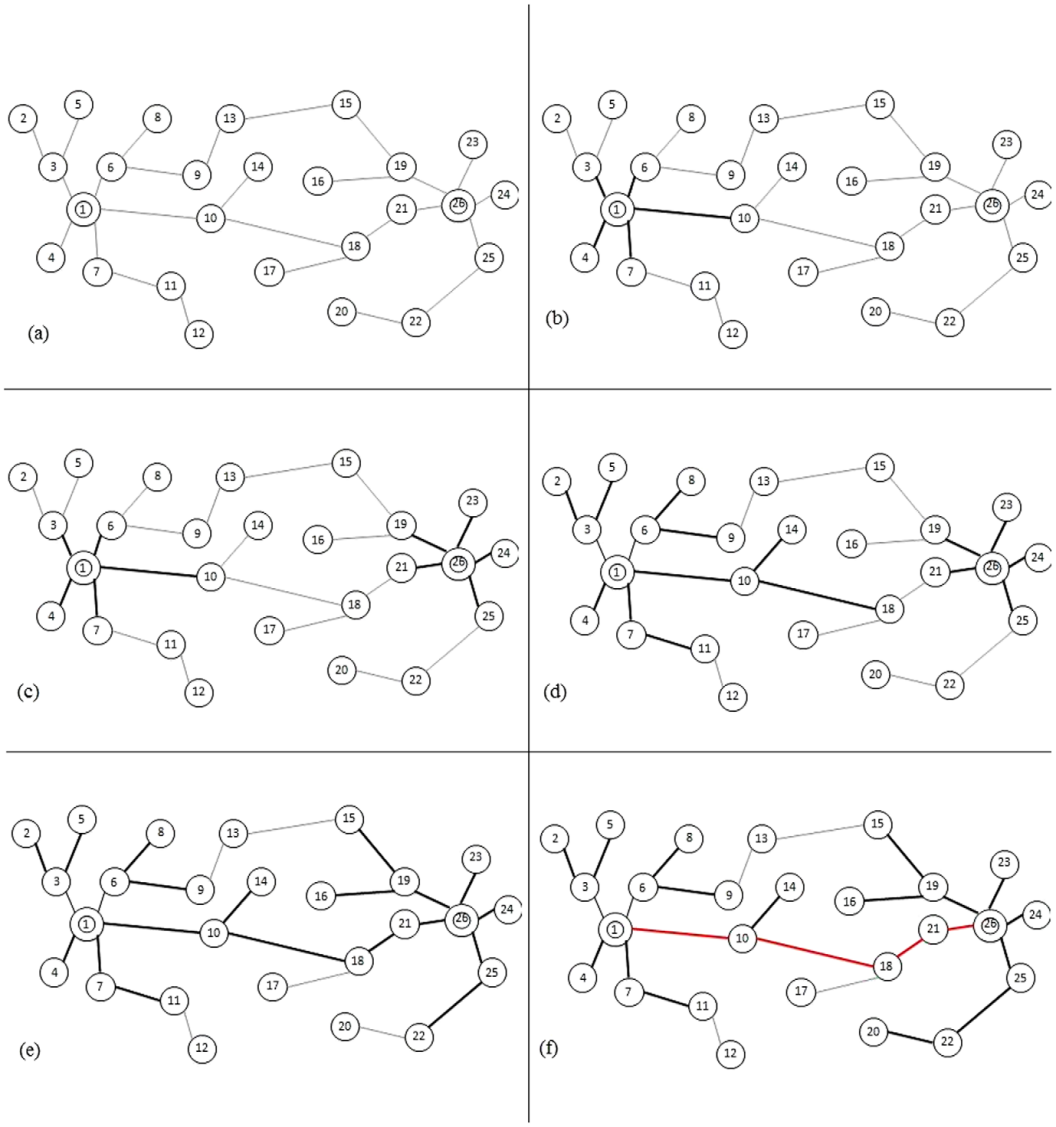
An intuitive example of the path finding algorithm.

More intuitively, search processes starts at the starting node and ending note at the same time. The process systematically explores all the neighboring nodes in sequence; then for each of those nearest neighboring nodes, it visits their unexplored neighbor nodes and records/updates all those stretching-out paths. The two processes end when they first explored the same node in the graph. Thus the shortest path is identified by combining the recorded path between the staring node and the coincidental node and between the coincidental node and the ending node. An example showing how the algorithm runs on Chem2Bio2RDF data are shown in Figure 3.

In the above example, we want to find the path between node 1 and node 26 (Figure 3-a):

1. Breadth First Search (BFS) explores the nearest neighbor of node 1 and it reaches node 3, 4, 6, 7, 10 (Figure 3-b);
2. Meanwhile, another BFS explores the nearest neighbor of node 26 similarly and it reaches node 19, 21, 23, 24, 25 (Figure 3-c);
3. Explore all the nearest neighbors of node 3, 4, 6, 7, 10, and it reaches 2, 5, 8, 9, 11, 14, 18 (Figure 3-d);
4. Meanwhile, explore all the nearest neighbors of node 19, 21, 22, 23, 24, 25, and it reaches 15, 16, 18, 22 (Figure 3-e);
5. One node (i.e., node 18) first gets visited by both BFS processes; algorithm ends. The shortest path between node 1 and node 26 is 1–10–18–21–26 (marked in red in Figure 3-f).

### 2.2.2 Near-Shortest Association Finding

In the previous step, we obtain the shortest association from each source entity to the target entity *v_j_*, including the shortest association with the length *L_min_* from the source entity *v_i_* to the target entity *v_j_*. In this step, based on the depth-first search, we try to find the near-shortest associations. The algorithm runs a straightforward *v_i_*-*v_j_* association enumeration algorithm (depth-first search). The depth-first search itself has an exponential complexity. We apply several strategies to reduce the computational cost. First we use an indicator *c*(*v*) to avoid loop in the association. Next we utilize the shortest associations d′(*v_i_*) found in Step 2 to prune the search space. Specifically, we extend an *v_i_*-*s* association to *u* along the relationship *e* = (*s*, *u*) if and only if d(*s*)+1+d′(*u*)<(1+*β*)*L_min_*, where d(*s*) is the length the current *v_i_*-*s* association and d′(*u*) is the shortest association from the entity *u* to the target entity *v_j_* (cf. line 11 in Figure 1).

Whenever an association *α*(*v_i_*, *v_j_*) is found using the above method, we calculate the length of the association d(*α*(*v_i_*, *v_j_*)) and add the association with its length to the association set *A*. The search terminates when no more association can be found. Then we rank all *α*(*v_i_*, *v_j_*)∈*A* with the lowest d(*α*(*v_i_*, *v_j_*)) on the top. Finally, we return the ranked associations. It is not easy to accurately analyze the complexity of the algorithm in this step. Depth-first search itself has an exponential complexity. However, in our algorithm we utilized several strategies to heuristically guide the search. The number of search steps is greatly reduced. An empirical analysis of the experimental results on the researcher network (with half million nodes and 2 millions edges) shows that the average search steps in this sub-process is 14,418 and the average time cost in this step is 0.34s which takes only 16.49% of the total time cost (about 3 seconds on average).

### 2.3 Bio-LDA

*Natural language processing (NLP) has been widely used to mine literatures in biomedical domain* [*37]*, [38**] *. Compared to traditional NLP techniques, which bases on linguistic rules of the documents, modern probabilistic models focus on the topical features of the documents. For example, LDA, a hierarchical Bayesian model, and its assorted variations, can* [*39]*, [40**] *capture groups of words that tend to be used to discuss the same topics. Applications of LDA in the biomedical domain have already produced promising results* [*22]*, [41], [42**] *. However, few of those applications take bio-terms (including genes, compounds, diseases, etc.) into a customized LDA model as the hidden variables. The Bio-LDA model used in this paper not only uncover the topical feature of common words, but more importantly, also the bio-terms. The similarity of bio-terms are then measured using KL-divergence, which, compared to the co-occurrence-based methods, is more helpful for identifying hidden associations* [*43]*, [44**] *.*

The Bio-LDA model extracts latent topics of bio-terms from biomedical literature, and which further provides semantically contextual evaluation for those associations identified by the path finding algorithm.

**Figure 4:**
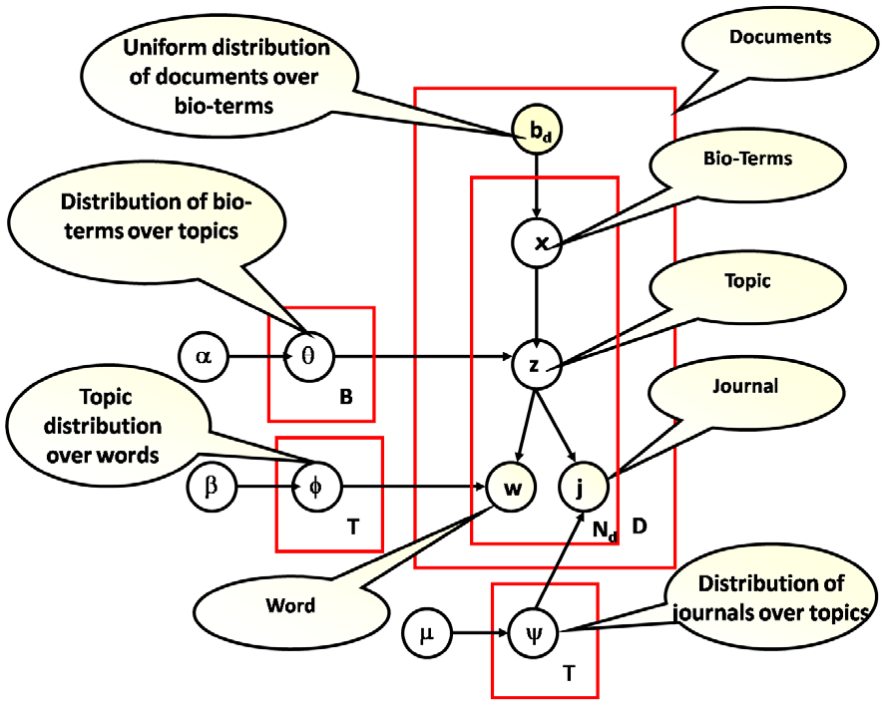
Graphical representation of the Bio-LDA. 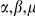
 are the Dirichlet priors for the distribution of bio-terms over topics, topic over words, and journals over topics. B is the total set of bio-terms. T denotes the total set of topics. D is the overall set of documents. *N_d_* is the set of words in a given document *d*.

Our Bio-LDA model extends the ACT model proposed by [45] as shown in Figure 4. Based on the results of Bio-LDA, we calculate entropy and KL divergence for any given two RDF nodes in the RDF graph to identity their semantic association.

The journal information is viewed as a stamp associated with each word in a paper. Intuitively, the co-occurrence of bio-terms in a document determines topics in this document and each topic then generates the words. 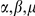 which are the Dirichlet priors for the distribution of bio-terms over topics, topic over words, and journals over topics. B is the total set of bio-terms. T denotes the total set of topics. D is the overall set of documents. *N_d_* is the set of words in a given document *d*.

The generative process can be summarized as follows:

1. For each topic *z*, draw *φ_z_* and *ψ_z_* respectively from Dirichlet priors *β_z_* and *μ_z_*;
2. For each word 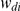 in paper *d*:
  - draw a bio-term 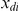 from *b*_d_ uniformly;
  - draw a topic 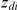 from a multinomial distribution 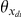

  - specific to bio-term 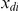, where 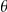 is generated from a Dirichlet prior 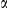;
  - draw a word 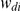 from multinomial 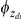 ;
  - draw a journal stamp 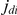 from multinomial 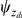.

In our model, Gibbs sampling is chosen for inference. As for the hyperparameters α, β, and μ, we take a fixed value (i.e., α = 50 = *T*, β = 0.01, and μ = 0.1). In the Gibbs sampling procedure, we first estimate the posterior distribution on just *x* and *z* and then use the results to infer θ,φ, and ψ. The posterior probability is calculated by the following equation:
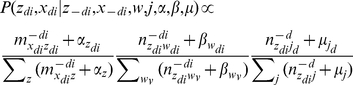
(1)where the superscript −*di* denotes a quantity, excluding the current instance (e.g., the *di*-th word token in the *d*-th paper). After Gibbs sampling, the probability of a word given a topic φ, the probability of a journal given a topic ψ, and the probability of a topic given a bio-term θ can be estimated as follows:
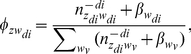
(2)

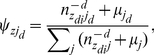
(3)

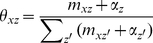
(4)

1. Bio-term Entropy over Topics

In information theory, entropy is a measure of the uncertainty associated with a random variable. It is also a measure of the average information content one is missing when one does not know the value of random variable. In our Bio-LDA model, we can compute the bio-term entropies over topics as shown in equation 5, which indicates that bio-terms tend to address a single topic or cover multiple topics. The higher the entropy is, the more diverse the bio-term is over topics.
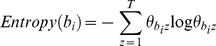
(5)

1. Semantic Association

Kullback-Leibler divergence (KL divergence) is a non-symmetric measure of the difference between two probability distributions. In our Bio-LDA model, we used the KL divergence as the non-symmetric distance measure for two bio-terms over topics, as shown in equation 6.
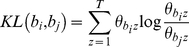
(6)The symmetric distance measure of two bio-terms over topics is the sum of two non-symmetric distances, as shown in equation 7.
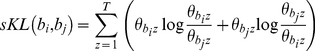
(7)


*sKL divergence measures the similarity between two probability distributions. In our Bio-LDA model, each bio-term is represented by a probability distribution which designates the strength of the semantic association between the bio-terms and a set of topics (or research issues). Thus sKL divergence is used to calculate the similarity between a pair of bio-terms by means of measuring the similarity between the two probability distributions associated with each bio-term of the pair. The smaller the sKL score is, the more semantically relevant the two bio-terms are in terms of their involvements with a set of research issues. This association score can combined with the pre-knowledge of bio-terms (i.e. Chem2Bio2Rdf) for novel knowledge discovery.* The score of a given directed semantic association is simply given by the accumulated distance between bio-terms on a path, as shown in equation 8. The score of an undirected path is given by the accumulated symmetric distance between bio-terms, as shown in equation 9. In this study, we do not evaluate the direction of the associations, focusing only on the association score calculated by the symmetric distances. The association search in Bio-LDA model is finding the associations with the smallest score.

## Results

We implemented the path finding algorithm described in section 2.2 using C++ and created a tool called *associationsearch* which will find paths of given length between any two items in our Chem2bio2rdf dataset. These items can be compounds, drugs, genes, pathways, diseases, or side-effects. These paths are then ranked (i.e., evaluated) by the Bio-LDA model described in section 2.3, and the user can select a maximum number of paths to return. The paths are then visualized using a flash interface within a browser.

We present two case studies that apply this method to address biological research problems.

### 3.1 Finding gene associations between thiazolinediones and cardiac side-effects

Insulin-sensitizing drugs from the thiozalinedione class have revolutionized the treatment of insulin-dependent diabetes yet have been beset by rare but serious side effects. The drugs Troglitazone, Rosiglitazone and Pioglitazone are thought to work by binding to the PPAR-gamma receptor, one of several nuclear receptors involved in fatty acid and glucose uptake. However, these receptors are also known to be involved in much larger scale regulation and metabolic processes including metabolism of xenobiotics (foreign substances in the body). Interference of some of these processes may be responsible for the side effects that have caused these drugs to “fall from grace”: Troglitazone was withdrawn from the U.S. market in 2000 due to adverse liver side effects; Rosiglitazone was until recently believed to be safe as it does not appear to have the hepatic side effects of Trogitazone, however it was restricted in the U.S. in 2011 and removed from the European market entirely in September 2010 due to increased risk of myocardial infarction in patients. Pioglitazone is currently under review.

**Figure 5:**
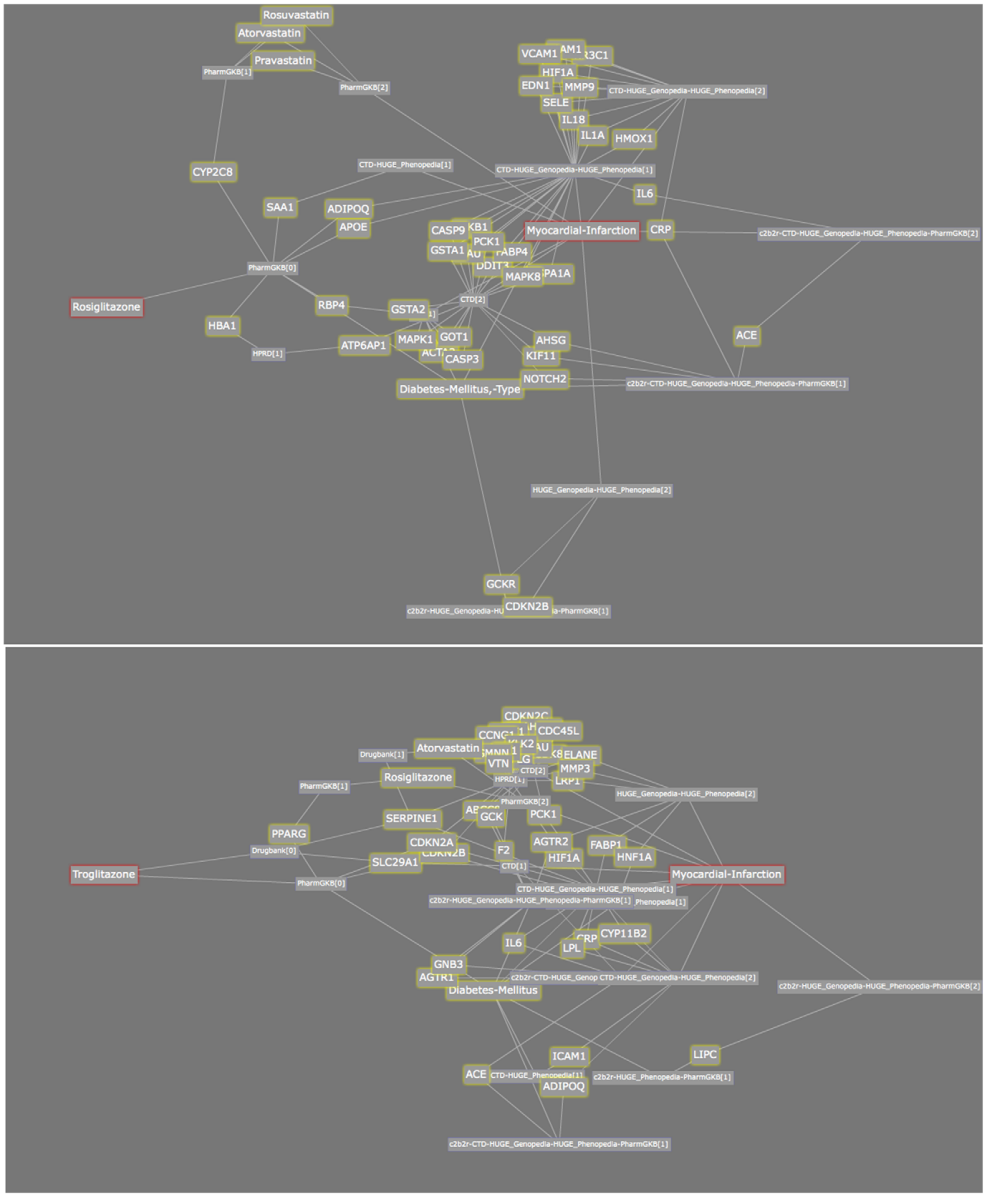
Ranked association graphs between myocardial infarction and Rosiglitazone (top) or Troglitazone (bottom) identify *SAA2*, *APOE*, *ADIPOQ*, and *CYP2C8* genes as significant for Rosiglitazone. *The red-outlined box is the starting node and ending node, that is, the bio-terms associations that we are searching for. Yellow-outlined boxes are the intermediate bio-terms. Other boxes indicate the types of the connection between the two intermediate bio-terms that it is connected to, which gives a hint on which database this connection is originated from.*

**Figure 6:**
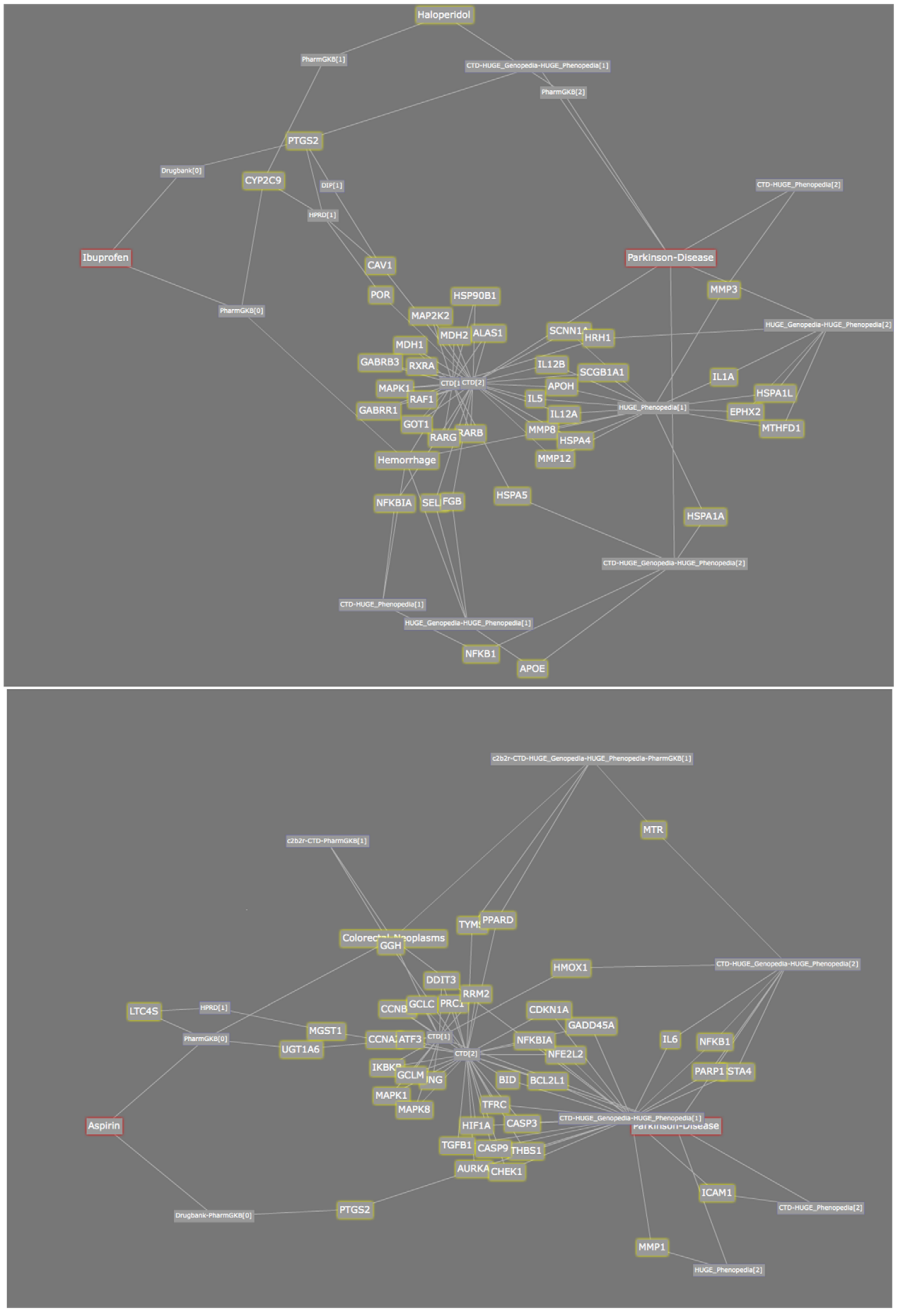
Ranked association graphs between Ibuprofen and Parkinson Disease (top) as well as Aspirin and Parkinson Disease. *The red-outlined box is the starting node and ending node, that is, the bio-terms associations that we are searching for. Yellow-outlined boxes are the intermediate bio-terms. Other boxes indicate the types of the connection between the two intermediate bio-terms that it is connected to, which gives a hint on which database this connection is originated from.*

We used our algorithms to examine ranked associations between Rosiglitazone and myocardial infarction, and Troglitazone and myocardial infarction, to see if we could identify gene associations that may account for the cardiac effects of Rosiglitazone. The association graphs for these two drugs are shown in Fig. 5. *The red-outlined box is the starting node and ending node, that is, the bio-terms associations that we are searching for. Yellow-outlined boxes are the intermediate bio-terms. Other boxes indicate the types of the connection between the two intermediate bio-terms that it is connected to, which gives a hint on which database this connection is originated from. Note that* *Fig. 5* *and* *Fig. 6* *are screenshots of the visualization provided by our application in which users can interactively moving the nodes and clicking the nodes to obtain more information about the node.* The graphs show that there is a strong ranked association between Rosiglitazone and myocardial infarction which is not present for Troglitazone, particularly involving four genes: *SAA2* (Serum Amyloid A 2), *APOE* (Apolipoprotein E), *ADIPOQ* (Adiponectin) and *CYP2C8* (Cytochrome P450 2C8). Examination of these genes indicates that all are involved in cardiovascular lipid metabolic processes. In particular, activation of *ADIPOQ* results in increased HDL (“good” cholesterol) and activation of *APOE* results in increased LDL levels (“bad” cholesterol), a potential mechanism that would account for Rosiglitazone's cardiac side effects as has recently been reported in the literature [46]. The next obvious question is whether Pioglitazone interacts with these genes. Association graphs between Pioglitazone and myocardial infarction (and Pioglitazone and Rosiglitazone) show strong associations between Pioglitazone and *ADIPOQ*, but not with *APOE*, indicating that Pioglitazone should increase HDLs but not LDLs. This is confirmed clinically by recent literature [45].

We further evaluated these relationships by directly examining the ranked paths from the BioLDA algorithm. Table 2 and 3 shows the symmetric KL divergence for semantic associations for the two pairs of bio-terms.

**Table 2:** Symmetric KL divergence for paths between Troglitazone and Myocardial infarction.

Path	sKL divergence
Troglitazone∼SLC29A1∼Dipyridamole∼Myocardial-infarction	16.151
Troglitazone∼Edema∼Iodixanol∼Myocardial-infarction	23.105
Troglitazone∼Edema∼Dipyridamole∼Myocardial-infarction	24.086
Troglitazone∼Congestive-heart-failure∼Bisoprolol∼Myocardial-infarction	24.151
Troglitazone∼Edema∼Bisoprolol∼Myocardial-infarction	24.744
Troglitazone∼PPARG∼Rosiglitazone∼Myocardial-infarction	25.454
Troglitazone∼Diabetes-Mellitus,-Type∼Benazepril∼Myocardial-infarction	25.732
Troglitazone∼Syncope∼Dipyridamole∼Myocardial-infarction	26.176
Troglitazone∼Hyperglycemia∼Rosiglitazone∼Myocardial-infarction	26.835
Troglitazone∼Syncope∼Bisoprolol∼Myocardial-infarction	26.953
Troglitazone∼Hyperglycemia∼Pioglitazone∼Myocardial-infarction	27.491
Troglitazone∼PPARG∼Pioglitazone∼Myocardial-infarction	28.126
Troglitazone∼Edema∼Nicardipine∼Myocardial-infarction	28.175
Troglitazone∼Edema∼Betaxolol∼Myocardial-infarction	28.564
Troglitazone∼Weight-gain∼Bisoprolol∼Myocardial-infarction	28.804
Troglitazone∼Edema∼Fosinopril∼Myocardial-infarction	29.052
Troglitazone∼Edema∼Amoxapine∼Myocardial-infarction	29.147
Troglitazone∼Edema∼Oxaprozin∼Myocardial-infarction	29.222
Troglitazone∼Malaise∼Betaxolol∼Myocardial-infarction	29.315
Troglitazone∼Edema∼Cilazapril∼Myocardial-infarction	29.361

**Table 3:** Symmetric KL divergence for paths of Rosiglitazone and Myocardial infarction.

Path	sKL divergence
Rosiglitazone∼Myocardial-infarction	17.231
Rosiglitazone∼Congestive-heart-failure∼Bisoprolol∼Myocardial-infarction	21.085
Rosiglitazone∼Heart-failure∼Bisoprolol∼Myocardial-infarction	22.067
Rosiglitazone∼Hyperglycemia∼Pioglitazone∼Myocardial-infarction	22.411
Rosiglitazone∼Hyperglycemia∼Cilazapril∼Myocardial-infarction	24.814
Rosiglitazone∼Hyperglycemia∼Betaxolol∼Myocardial-infarction	24.892
Rosiglitazone∼Hypoglycemia∼Bisoprolol∼Myocardial-infarction	25.269
Rosiglitazone∼Hyperglycemia∼Oxaprozin∼Myocardial-infarction	25.494
Rosiglitazone∼Hyperglycemia∼Diazoxide∼Myocardial-infarction	25.767
Rosiglitazone∼Bilirubinemia∼Eletriptan∼Myocardial-infarction	26.273
Rosiglitazone∼Bilirubinemia∼Dolasetron∼Myocardial-infarction	26.439
Rosiglitazone∼Diabetes-Mellitus,-Type∼Benazepril∼Myocardial-infarction	26.634
Rosiglitazone∼Hyperglycemia∼Bosentan∼Myocardial-infarction	26.683
Rosiglitazone∼Hyperglycemia∼Candesartan∼Myocardial-infarction	26.688
Rosiglitazone∼Hyperglycemia∼Quinapril∼Myocardial-infarction	27.381
Rosiglitazone∼Diabetes-Mellitus∼Pioglitazone∼Myocardial-infarction	27.821
Rosiglitazone∼Hypoglycemia∼Betaxolol∼Myocardial-infarction	27.832
Rosiglitazone∼Hypoglycemia∼Pioglitazone∼Myocardial-infarction	28.316
Rosiglitazone∼Dizziness∼Bisoprolol∼Myocardial-infarction	28.681
Rosiglitazone∼Nasopharyngitis∼Bosentan∼Myocardial-infarction	28.699

### 3.2 Associations between non-steroidal anti-inflammatory drugs (NSAIDs), inflammation and Parkinson Disease

Recent research [47] has shown that use of Ibuprofen, a non-steroidal anti-inflammatory drug, is clinically associated with reduced risk of Parkinson Disease. This effect is not found with other painkillers, such as Aspirin and Acetaminophen (Paracetamol). It is speculated that this effect may be due to the anti-inflammatory effects of Ibuprofen on neuroinflammation. We performed searches to (i) identify paths containing genes linking Ibuprofen, inflammation and Parkinson Disease (through three searches – Ibuprofen-Parkinson Disease, Ibuprofen-inflammation and inflammation-Parkinson Disease) and (ii) identify genes associated with Ibuprofen but not with the other NSAIDS (in case this could be used to account for the differential activity with Aspirin, etc). Our searching identified 70 genes that are associated with Ibuprofen, inflammation and Parkinson Disease, 9 of which are known to be linked to inflammation: *IL1A*, *IL1B*, *IL1RN*, *IL6*, *LTA*, *NFKB1*, *NFKBIA*, *PTGS2* and *TNF*.

Of particular note, these searches identified a clear direct connection between the primary target of Ibuprofen (*PTGS2*, or *Cox2* – Ibuprofen is a nonspecific inhibitor that also targets *Cox1*), and Parkinson Disease. This link maps to experimental data in the CTD dataset. The *Cox2* link is supported by a variety of recent research [48]–[52] which indicates that neuroinflammation is implicated in Parkinson's Disease, and that the *Cox2* gene is implicated in this inflammation process. Indeed, selective and nonselective *Cox2* inhibitors have been examined for their effect in this inflammatory process [52]. Selective Cox2 inhibitors may be of particular interest.

In our second search, we found a single gene, *AMBP*, which is differentially associated with Ibuprofen (and not with other NSAIDS), and which is associated with Parkinson disease (but not inflammation), based on a 1996 study which showed the potential of *AMBP* as a biomarker for the disease [53]. Several of the results searches are shown in Figure 6. *The red-outlined box is the starting node and ending node, that is, the bio-terms associations that we are searching for. Yellow-outlined boxes are the intermediate bio-terms. Other boxes indicate the types of the connection between the two intermediate bio-terms that it is connected to, which gives a hint on which database this connection is originated from.*

We further evaluated these relationships by directly examining the ranked paths from the BioLDA algorithm. Table 4 and 5 shows the symmetric KL divergence for semantic associations for the two pairs of bio-terms. The smaller the KL divergence is, the more thematically similar the bioterms along the path are in the literature. In Table 4, the path Ibuprofen-*PTGS2*-PD ranks high. Teismann et al. [54] studied the relationship between *COX-2*(*PTGS2*) and Parkinson Disease by MPTP (1-methyl-4-phenyl-1,2,3,6-tetrahydropyridine) model. MPTP induces Parkinson Disease and *COX-2*. The authors claimed that *COX-2* inhibitors may be therapies for Parkinson Disease if the inhibitors have ability to penetrate the blood brain barrier. Many paths that connect Ibuprofen and Parkinson Disease through Hemorrhage and other genes have shown small KL divergence. Several studies have shown that Ibuprofen is helpful in preventing or decreasing susceptibility to different types of hemorrhage [55]–[58].

**Table 4:** Symmetric KL divergence for paths between Ibuprofen and Parkinson Disease.

*Paths*	*KL*
Ibuprofen PharmGKB CYP2C9 HPRD POR CTD Parkinson-Disease	28.077
Ibuprofen Drugbank PTGS2 CTD-HUGE_Genopedia-HUGE_Phenopedia Parkinson-Disease	33.049
Ibuprofen PharmGKB Hemorrhage HUGE_Phenopedia HSPA1L HUGE_Genopedia-HUGE_Phenopedia Parkinson-Disease	36.573
Ibuprofen PharmGKB CYP2C9 PharmGKB Haloperidol PharmGKB Parkinson-Disease	37.339
Ibuprofen PharmGKB Hemorrhage CTD GABRR1 CTD Parkinson-Disease	37.791
Ibuprofen PharmGKB Hemorrhage HUGE_Phenopedia HSPA4 CTD Parkinson-Disease	37.842
Ibuprofen PharmGKB Hemorrhage CTD HRH1 HUGE_Genopedia-HUGE_Phenopedia Parkinson-Disease	38.153
Ibuprofen PharmGKB Hemorrhage CTD RARG CTD Parkinson-Disease	38.558
Ibuprofen PharmGKB Hemorrhage CTD MAP2K2 CTD Parkinson-Disease	38.858
Ibuprofen PharmGKB Hemorrhage CTD HSPA5 CTD-HUGE_Genopedia-HUGE_Phenopedia Parkinson-Disease	39.055
Ibuprofen PharmGKB Hemorrhage HUGE_Phenopedia MMP8 CTD Parkinson-Disease	39.668
Ibuprofen PharmGKB Hemorrhage HUGE_Phenopedia SCNN1A CTD Parkinson-Disease	39.783
Ibuprofen PharmGKB Hemorrhage CTD GOT1 CTD Parkinson-Disease	39.896
Ibuprofen PharmGKB Hemorrhage HUGE_Phenopedia HSPA1A CTD-HUGE_Genopedia-HUGE_Phenopedia Parkinson-Disease	40.331
Ibuprofen PharmGKB Hemorrhage CTD HSP90B1 CTD Parkinson-Disease	40.886
Ibuprofen PharmGKB Hemorrhage HUGE_Phenopedia IL1A HUGE_Genopedia-HUGE_Phenopedia Parkinson-Disease	41.056
Ibuprofen PharmGKB Hemorrhage HUGE_Phenopedia MMP12 CTD Parkinson-Disease	41.127
Ibuprofen PharmGKB Hemorrhage HUGE_Genopedia-HUGE_Phenopedia SELP CTD Parkinson-Disease	41.278
Ibuprofen PharmGKB Hemorrhage CTD RARB CTD Parkinson-Disease	41.455
Ibuprofen PharmGKB Hemorrhage HUGE_Phenopedia SCGB1A1 CTD Parkinson-Disease	41.47

**Table 5:** Symmetric KL divergence for paths between Aspirin and Parkinson Disease.

*Paths*	*KL*
Aspirin PharmGKB Colorectal-Neoplasms CTD-HUGE_Genopedia-HUGE_Phenopedia CHEK1 CTD Parkinson-Disease	25.682
Aspirin PharmGKB UGT1A6 CTD Parkinson-Disease	26.031
Aspirin PharmGKB Colorectal-Neoplasms CTD-HUGE_Genopedia-HUGE_Phenopedia CASP9 CTD Parkinson-Disease	26.771
Aspirin PharmGKB Colorectal-Neoplasms CTD IKBKB CTD Parkinson-Disease	27.084
Aspirin PharmGKB Colorectal-Neoplasms CTD-HUGE_Genopedia-HUGE_Phenopedia NFKB1 CTD-HUGE_Genopedia-HUGE_Phenopedia Parkinson-Disease	27.437
Aspirin PharmGKB LTC4S HPRD MGST1 CTD Parkinson-Disease	27.678
Aspirin PharmGKB Colorectal-Neoplasms CTD DDIT3 CTD Parkinson-Disease	27.919
Aspirin PharmGKB Colorectal-Neoplasms CTD CCNB2 CTD Parkinson-Disease	27.979
Aspirin PharmGKB Colorectal-Neoplasms CTD-HUGE_Genopedia-HUGE_Phenopedia TFRC CTD Parkinson-Disease	28.226
Aspirin PharmGKB Colorectal-Neoplasms CTD-HUGE_Genopedia-HUGE_Phenopedia GSTA4 CTD-HUGE_Genopedia-HUGE_Phenopedia Parkinson-Disease	28.416
Aspirin PharmGKB Colorectal-Neoplasms c2b2r-CTD-HUGE_Genopedia-HUGE_Phenopedia-PharmGKB MTR CTD-HUGE_Genopedia-HUGE_Phenopedia Parkinson-Disease	29.2
Aspirin PharmGKB Colorectal-Neoplasms CTD-HUGE_Genopedia-HUGE_Phenopedia NFE2L2 CTD Parkinson-Disease	29.642
Aspirin PharmGKB Colorectal-Neoplasms CTD CCNA2 CTD Parkinson-Disease	29.669
Aspirin PharmGKB Colorectal-Neoplasms CTD RRM2 CTD Parkinson-Disease	30.126
Aspirin PharmGKB Colorectal-Neoplasms CTD-HUGE_Genopedia-HUGE_Phenopedia TGFB1 CTD Parkinson-Disease	30.249
Aspirin PharmGKB Colorectal-Neoplasms CTD-HUGE_Genopedia-HUGE_Phenopedia BCL2L1 CTD Parkinson-Disease	30.357
Aspirin PharmGKB Colorectal-Neoplasms CTD GCLM CTD Parkinson-Disease	30.386
Aspirin PharmGKB Colorectal-Neoplasms CTD MAPK8 CTD Parkinson-Disease	30.58
Aspirin PharmGKB Colorectal-Neoplasms CTD-HUGE_Genopedia-HUGE_Phenopedia HIF1A CTD Parkinson-Disease	30.805
Aspirin PharmGKB Colorectal-Neoplasms CTD-HUGE_Genopedia-HUGE_Phenopedia CHEK1 CTD Parkinson-Disease	25.682

## Discussion

In this paper, we propose a scalable path finding algorithm and a topic model called Bio-LDA so as to mine semantic associations in integrated platform of various biomedical databases. The path finding algorithm can identify semantic paths between any two classes or instances in the linked open data in the biomedical domain. The Bio-LDA model extracts distributions of topics for bio-entities, which can provide topic-sensitive ranking of identified semantic associations. The two use cases presented in the paper demonstrate the rich possibilities that the proposed algorithm and model can contribute to crucial issues in biomedical domain, including Polypharmacology, drugs related to inhibition of a certain gene involved in diseases, and drug-like compounds. *The application discussed in this paper is made available through* http://cheminfov.informatics.indiana.edu:8080/yuysun/hychembiospace.html.

Our path finding algorithm can be readily applied to an extensible network of linked open data both in the biomedical domain and other domains. In addition, based on the Bio-LDA model, we calculate the entropy and KL divergence for genes, compounds and diseases in the paths. The entropy shows to what extent the bio-terms are involved in multiple topics among biomedical literature; the KL divergence indicates the similarity between two bio-terms involved with different topics. Values extracted from another knowledge base (Medline) can be further integrated with user preferences to assign weight to semantic associations or to rank semantic associations. We also adopt expert and literature investigation to assess the result and value of the proposed algorithm, which indicates the algorithm can help discover invisible knowledge and identify potential research issues by obtaining and integrating existing knowledge.

For future work, we plan to further explore the potential of using the knowledge extracted through topic mining model to rank semantic associations. Moreover, we plan to design a parallel implementation of Bio-LDA and semantic association finding algorithm on MPI and MapReduce, which smoothes out storage and computation bottlenecks. Meanwhile, we would also like to establish an interactive searching system for semantic associations based on Chem2Bio2RDF database and extend our algorithm to incorporate heuristics from user preferences, context, or domain-specific rules.
